# Health information-seeking behavior among women with polycystic ovary syndrome: A scoping review protocol

**DOI:** 10.1371/journal.pone.0342690

**Published:** 2026-02-23

**Authors:** Xia Chen, Xiaoping Xie, E. Tan, Yingni Liang, Fen Liu, Zhongyu Li, Yinhua Su

**Affiliations:** 1 School of Nursing, Hengyang Medical School, University of South China, Hengyang, Hunan, China; 2 The First Affiliated Hospital, University of South China, Hengyang, Hunan, China; Kasr Alainy Medical School, Cairo University, EGYPT

## Abstract

**Background:**

Polycystic ovary syndrome (PCOS) is the most common endocrine metabolic disorder among women of childbearing age, and self-management of PCOS patients relies on their ability to obtain health information. The proliferation of digital technologies, particularly social media and health applications, has fundamentally transformed health information-seeking behaviors (HISB) in this population. However, the present information behavior patterns of PCOS patients have not yet been systematically integrated.

**Objectives:**

This scoping review aims to systematically map the landscape of HISB in women with PCOS by utilizing Wilson’s model of information-seeking behavior as theoretical framework. It seeks to synthesize evidence on their information needs, preferred channels, behavioral types, and key influencing factors.

**Methods:**

The scoping review will adhere to Arksey and O’Malley’s methodological framework and report following the Preferred Reporting Items for Systematic Reviews and Meta-Analyses (PRISMA) guidelines. The review will include English-language literature published from inception up to November 30, 2025, searched through PubMed, Web of Science, Embase (Ovid), CINAHL, Cochrane Library, and APA PsycINFO. To find more relevant studies, we will also search grey literature, the reference lists of the included studies, and related systematic reviews. Two researchers will independently screen titles/abstracts, followed by full-text articles, to assess whether articles meet the inclusion criteria. A third researcher will resolve any discrepancies. Data extraction and narrative synthesis will be structured around the core constructs of Wilson’s model, providing a theory-informed analysis of the evidence.

**Ethics and dissemination:**

Since this review involves collecting data from existing literature and does not involve human participants, ethical approval is not required. This scoping review will be submitted for publication to a peer reviewed academic journal.

## Introduction

Polycystic Ovary Syndrome (PCOS) is the most common endocrine and metabolic disorder among women of reproductive age, with a global prevalence ranging from 5% to 18% [1]. This condition presents with heterogeneous clinical manifestations, such as menstrual irregularities, hyperandrogenism, and the presence of polycystic ovaries [2], and is associated with long-term reproductive, metabolic, and psychological complications [3–5]. Given this complex clinical profile, effective self-management strategies become essential for mitigating disease complications and optimizing quality of life, which are fundamentally dependent on appropriate information-seeking behaviors [6,7].

Health information-seeking behavior (HISB) refers to the process in which individuals actively acquire, evaluate, and utilize health-related information to meet their health needs, involving multidimensional dynamic interactions such as information source selection, behavior patterns, and influencing factors [8]. However, existing studies reveal some persistent challenges in the HISB of PCOS patients. Firstly, the uneven quality of information from healthcare providers can lead to confusion in patient decision-making [9,10]; inaccurate or irrelevant information overload can harm patient healthcare, health outcomes, and doctor-patient relationships [11,12]. These factors collectively compromise the reliability and effectiveness of the health information ecosystem of PCOS patients.

While extensive research has characterized HISB patterns in other chronic conditions like diabetes and cancer [13,14], a comprehensive understanding of HISB in PCOS remains elusive. Existing syntheses have provided valuable but fragmented insights. Several systematic reviews have focused on the information needs of women with PCOS [15,16], a focus that risks overlooking the information sources and related factors. Gibson Holm [15] pointed out that women with PCOS will obtain relevant information through the Internet, but there is little in-depth research on the types of websites used by women and their most practical functions. A scoping review on polycystic ovary syndrome and IoT technology [17] explored the application of six IoT fields in self-management of PCOS patients, and found that IoT technology has the potential to assist PCOS patients in symptom tracking, information acquisition, and self-management. However, this review primarily emphasized technological aspects while only briefly addressing information needs, without a thorough exploration of HISB patterns and influencing factors.

This fragmented approach reveals a critical gap: the absence of a unified and systematic mapping of the entire spectrum of HISB in PCOS. To date, no study has concurrently and systematically investigated the interplay between what information patients need (needs), where they look for it (sources and channels), how they engage in the search process (behavioral types), and what personal or environmental factors enable or hinder these behaviors (determinants).

To address this gap, this scoping review will employ Wilson’s model [18] as an overarching theoretical framework. This model provides a comprehensive structure for organizing and connecting key concepts related to information needs, seeking behaviors, and use, making it ideally suited to capture the multifaceted nature of HISB in PCOS. The application of this established model ensures a rigorous and theory-informed synthesis by using the model’s constructs to guide the analysis and presentation of the evidence.

Thus, an overview of the HISB of women with PCOS patterns is urgently required to establish evidence-based guidelines for clinical practice and future research, ensuring optimal information support for this population. The study aims to (1) investigate the current status of HISB in PCOS patients, (2) reveal the information and methodological characteristics of HISB, and (3) identify key determinants influencing these behaviors.

## Methods

### Design

The scoping review will adhere to Arksey and O’Malley’s methodological framework [19] and report following the Preferred Reporting Items for Systematic Reviews and Meta-Analyses (PRISMA) guidelines [20] (see S1 Checklist). Specifically, it will consist of five phases: (1) defining the research question; (2) identifying relevant studies; (3) selecting studies; (4) graphing the data; and (5) collating, summarizing, and reporting the results. This protocol has been registered with the Open Science Framework under DOI: https://doi.org/10.17605/OSF.IO/SC9KX/SC9KX. Ethical approval was not required, as this review involved only the analysis of previously published literature and did not include human participants.

### Identifying the research questions

The scoping review aims to provide a comprehensive review of HISB of women with PCOS and propose recommendations for future research. By consulting and discussing with the research team, the main questions are as follows:

(1) What are the health-related information needs of PCOS patients?
(2) What are the channels for PCOS patients to seek health information?
(3) What are the types of health information-seeking behaviors among PCOS patients?
(4) What factors are related to the HISB of women with PCOS?

### Theoretical framework

This scoping review will be guided by Wilson’s model [18], which provides a comprehensive framework for understanding health information seeking, depicting a cyclical process from information demand to use. It identifies four different types of information-seeking behavior: passive attention (unintentional information exposure), passive search (serendipitous information discovery during unrelated queries), active search (purposeful information seeking behavior identified as the primary focus of this investigation), and ongoing search (systematic information updating). Additionally, Wilson classified intervention variables into five distinct dimensions: (1) psychological factors, (2) demographic attributes, (3) role-related/interpersonal influences, (4) environmental constraints, and (5) source characteristics.

### Identifying relevant studies

#### Inclusion criteria.

The included articles must be written in English as we did not have resources to support translation. This is a limitation of the study as it may result in the exclusion of relevant articles in other languages. The search criteria will include articles published from inception up to November 30, 2025, which was characterized by significant advancements in information technology, including the proliferation of mobile devices and the rise of social media [21]. We adopted the PCC (population, concept, context) framework, following the JBI guidelines for scoping reviews [22] to determine further eligibility criteria (see below).

#### Participants.

We included studies of people who were aged 18 years and above and self-reported or were diagnosed with PCOS. Studies that focus on the perspective of healthcare providers or information needs have been excluded.

#### Concept.

We included studies focusing on the information needs, information sources, and information-seeking behavior of women with PCOS. Information needs [23] refer to the requirements of patients with polycystic ovary syndrome to understand, master, and utilize physiological, social, and psychological information during disease diagnosis, treatment, and self-management. Information sources are defined as various channels or carriers through which individuals obtain information in the process of health decision-making. When PCOS patients have information needs, they often bridge the information gap through channels such as healthcare providers, social media, and the Internet.

This review will consider all articles that emphasize the information needs, information sources, and information-seeking behaviors of women with polycystic ovary syndrome.

#### Context.

Articles describing the information needs, sources, or seeking behaviors of PCOS patients will be included.

#### Exclusion criteria.

Studies were excluded due to any one of the following reasons: (1) commentary, opinion, or viewpoint, editorial; (2) only published in abstract form, unable to obtain the full text; (3) HISB focused on healthcare professionals (HCPs); (4) research with missing data or unavailable full text.

### Search strategy

Our search strategy combined the mesh subject headings (MeSH) and a list of free text terms, including “polycystic ovary syndrome,” “PCOS,” “information needs,” “information sources,” “information seeking behavior,” “information preferences,” and “patient education.” We conducted a detailed search across the following databases, such as PubMed, Web of Science, Embase (Ovid), CINAHL, Cochrane Library, and APA PsycINFO. Specific search terms were modified for each database (Table 1). The Google search engine will be used to search for editorials, reports from governments, international organizations, professional associations, and grey literature. In addition, we will manually search the reference list, subsequent citations, and any latest publications that have been accepted and are accessible online.

**Table 1:** Pubmed search strategy. Note: This example search using Pubmed was run on 11/30/2025; Language: English.

Search	Query	Records retrieved
**#1**	((((((Polycystic Ovary Syndrome[MeSH Terms]) OR (Polycystic Ovarian Syndrome[Title/Abstract])) OR (Sclerocystic Ovarian Degeneration[Title/Abstract])) OR (Sclerocystic Ovary Syndrome[Title/Abstract])) OR (Stein-Leventhal Syndrome[Title/Abstract])) OR (Sclerocystic Ovaries[Title/Abstract])) OR (PCOS[Title/Abstract])	26,675
**#2**	(((((((((((information need*[Title/Abstract]) OR (health information need[Title/Abstract])) OR (patient need*[Title/Abstract])) OR (Educational Need*[Title/Abstract])) OR (Patient Education[Title/Abstract])) OR (Education of Patients[Title/Abstract])) OR (Consumer Health Information[Title/Abstract])) OR (psychological need*[Title/Abstract])) OR (Supportive need*[Title/Abstract])) OR (healthcare need*[Title/Abstract])) OR (Mental health need[Title/Abstract])) OR (need*[Title/Abstract])	2,894,622
**#3**	((((information source*[Title/Abstract]) OR (source* of information[Title/Abstract])) OR (information search*[Title/Abstract])) OR (information seeking[Title/Abstract])) OR (Information Seeking Behavior*[Title/Abstract])	33,834
**#4**	#1 AND (#2 OR #3)	2526

### Study selection

When we finish the search process, the citations will be exported from electronic search interfaces to EndNote 20, a reference management software that aims to eliminate duplication and screening. EndNote will also be employed to generate the reference list for the review. To ensure consistent and objective screening, two reviewers will independently assess the titles and abstracts of all retrieved records against predefined criteria. Before formal screening, a calibration exercise using 30 randomly selected records will be conducted to align reviewers’ judgments. Records passing initial screening will undergo full-text review by the same two reviewers for final eligibility determination. Any disagreements regarding study eligibility will first be resolved through discussion. If consensus is not reached, a third senior reviewer will arbitrate and make the final decision. For studies unavailable through standard channels, the corresponding author will be contacted by email. If the full text remains unobtainable after two weeks, the study will be excluded and recorded as “unretrievable,”with reasons documented. A separate screening log maintained in Microsoft Excel will record specific exclusion reasons for each study excluded at the full-text stage, consistent with predefined criteria. This ensures full documentation and auditability. The search and selection process will be fully reported in the final scoping review and illustrated with a PRISMA flow diagram [24] (S1 Fig).

### Charting the data

Following the study selection, a collaborative and phased data extraction process will be implemented. First, the research team will jointly develop a standardized data extraction form using Microsoft Excel. To ensure clarity, completeness, and reliability, the form will be piloted by two independent reviewers on five randomly selected included studies and further refined based on their feedback. Subsequently, the two reviewers will independently extract data from all included studies using the finalized form. The data to be extracted include basic descriptive information of each study, as well as specific details related to the research questions and the dimensions of Wilson’s model (see Tables 2–6).

**Table 2:** Data extraction.

Ariticle	Title	Author(s)	Country	Year of publication	Study purpose	Study design/method	General condition of the patients
1							
2							
3							

**Table 3:** Information needs categories of women with PCOS.

Main category	Subcategory	Number of Studies	Studies
			

**Table 4:** The current and preferred sources of information.

Information Sources		Number of Studies	Studies
Current			
Perferred			

**Table 5:** The types of health information-seeking behavior.

Types	Examples	Studies
Passive attention		
Passive search		
Active search		
Ongoing search		
Avoid seeking		

**Table 6:** Associated factors of HISB among women with PCOS.

Associated variables	Number of Studies	Studies
Demographic		
Psychological		
Role-related or interpersonal		
Environmental		
Source-related characteristics		
Credibility		
Usefulness		
Disease-related		

All independently extracted data will undergo systematic cross-verification. Any discrepancies identified will first be discussed and resolved through consultation between the two reviewers. If consensus cannot be reached, a third senior reviewer will be consulted for arbitration to ensure data accuracy and consistency in the final conclusions.

### Summarizing, collating, and reporting the results

Data synthesis will be conducted in accordance with the JBI (Joanna Briggs Institute) methodology for scoping reviews [20], utilizing a narrative synthesis approach. Quantitative data (e.g., study characteristics) and qualitative data (e.g., content relevant to the review questions) will be analyzed separately and presented in parallel to systematically map the existing evidence. The synthesis will be structured around the key constructs of Wilson’s model to enable a coherent exploration of health information-seeking behaviors among women with PCOS.

Quantitative data pertaining to study characteristics, such as publication year, country of origin, and study design, will be summarized using descriptive statistics (frequencies and percentages). These summaries will be presented in tables and charts to clearly illustrate the distribution and profile of the included evidence.

For qualitative data related to information needs, seeking behaviors, and influencing factors, reflexive thematic analysis following the six-phase framework by Braun and Clarke will be employed. This analytical approach allows for an in-depth examination of patterns and themes within the data. The analysis will be supported by NVivo® qualitative data analysis software and will proceed through the following stages [25]: i) repeated reading and annotation of the data to achieve familiarity; ii) open coding to develop an initial coding framework based on emergent categories; iii) systematic review of codes to organize them into potential themes; iv) refinement and revision of preliminary themes; v) exploration of relationships among codes to further define and refine the themes; and vi) systematic writing-up of the findings.

All results will be presented in tabular and graphical formats, accompanied by a narrative summary that explicitly links the findings to the objectives and research questions of the review.

## Discussion

This scoping review will provide a broad overview of the health information-seeking behavior among women with PCOS. Until now, many reviews have focused on a single aspect, such as information needs rather than the more holistic approach proposed here. This protocol is the first to plan the use of Wilson’s model as a structured framework to guide data extraction and synthesis. This will ensure a systematic and consistent approach to capturing all key components of information behavior—information needs, sources, types of seeking behavior, and influencing factors—across the included literature. The results will be presented narratively and in tabular form, organized by these core constructs of Wilson’s model, to provide a coherent and comprehensive overview.

A potential limitation of this protocol is the inclusion of English-language literature only. We made this decision for practical reasons, to ensure consistency and accuracy during the screening and data extraction process, given the research team’s language capabilities. We acknowledge that this might limit the cultural scope of our findings, and we will explicitly discuss this limitation in the full review. Additionally, as a scoping review designed to map the literature, this study does not include a formal appraisal of the methodological quality or risk of bias in the included studies.

In carrying out this work, we hope to achieve several practical outcomes. First, by introducing and validating Wilson’s model, we provide a structured framework for future research on PCOS, enabling more consistent and comparable studies on information behavior. More importantly, the findings are intended to have direct practical applicability: (1) In clinical settings, they can help healthcare professionals tailor patient education and counseling to address the most common information sources, needs, and barriers identified. (2) For patient support, the results can guide the development of more targeted and trustworthy information resources that match how and where women actually seek information. (3) On a systemic level, uncovering patterns of information access and barriers can inform health policy and resource allocation to improve health literacy and equity. Ultimately, this review seeks to empower women with PCOS by bridging the gap between their information-seeking experiences and the resources available, ensuring that future research and clinical practice are informed by a comprehensive, patient-centered evidence base.

## Supporting information

S1 Checklist PRISMA checklist. (DOCX)

S1 Fig PRISMA flow diagram. (DOCX)
